# Acupuncture treatment vs. cognitive rehabilitation for post-stroke cognitive impairment: A systematic review and meta-analysis of randomized controlled trials

**DOI:** 10.3389/fneur.2023.1035125

**Published:** 2023-02-09

**Authors:** Yang Liu, Fuyan Chen, Peng Qin, Lu Zhao, Xingping Li, Jiangqin Han, Zi Ke, Honghang Zhu, Bangqi Wu

**Affiliations:** ^1^Department of Acupuncture, First Teaching Hospital of Tianjin University of Traditional Chinese Medicine, Tianjin, China; ^2^National Clinical Research Center for Chinese Medicine Acupuncture and Moxibustion, Tianjin, China

**Keywords:** acupuncture treatment, post-stroke cognitive impairment, meta-analysis, cognitive rehabilitation, non-pharmacological treatment

## Abstract

**Background:**

Cognitive impairment is one of the common sequelae after stroke, which not only hinders the recovery of patients but also increases the financial burden on families. In the absence of effective therapeutic measures, acupuncture treatment has been widely used in China to treat post-stroke cognitive impairment (PSCI), but the specific efficacy is unclear. Therefore, this review aimed to evaluate the true efficacy of acupuncture treatment in patients with PSCI.

**Methods:**

We searched eight databases [PubMed, Embase, Web of Science, Cochrane Central Register of Controlled Trials, China Biomedical Literature Database (CBM), China Science and Technology Journal (VIP) database, the China National Knowledge Infrastructure (CNKI) database, and Wan fang database] from the inception to May 2022 for randomized controlled trials (RCTs) related to acupuncture treatment combined with cognitive rehabilitation (CR) for PSCI. Two investigators independently used a pre-designed form to extract valid data from eligible RCTs. The risk of bias was assessed through tools provided by the Cochrane Collaboration. The meta-analysis was implemented through Rev Man software (version 5.4). The strength of the evidence obtained was evaluated using GRADE profiler software. Adverse events (AEs) were collected by reading the full text and used to evaluate the safety of acupuncture treatment.

**Results:**

Thirty-eight studies involving a total of 2,971 participants were included in this meta-analysis. Overall, the RCTs included in this meta-analysis were poor in methodological quality. The combined results showed that acupuncture treatment combined with CR showed significant superiority compared to CR alone in terms of improving cognitive function [Mean Difference (MD) = 3.94, 95% confidence intervals (CI): 3.16–4.72, *P* < 0.00001 (MMSE); MD = 3.30, 95%CI: 2.53–4.07, *P* < 0.00001 (MoCA); MD = 9.53, 95%CI: 5.61–13.45, *P* < 0.00001 (LOTCA)]. Furthermore, the combination of acupuncture treatment and CR significantly improved patients' self-care ability compared to CR alone [MD = 8.66, 95%CI: 5.85–11.47, *P* < 0.00001 (MBI); MD = 5.24, 95%CI: 3.90–6.57, *P* < 0.00001 (FIM)]. Meanwhile, subgroup analysis showed that MMSE scores were not sufficiently improved in the comparison of electro-acupuncture combined with CR versus CR alone (MD = 4.07, 95%CI: −0.45–8.60, *P* = 0.08). However, we also observed that electro-acupuncture combined with CR was superior to the use of CR alone in improving MoCA and MBI scores in patients with PSCI [MD = 2.17, 95%CI: 0.65–3.70, *P* = 0.005 (MoCA); MD = 1.74, 95%CI: 0.13–3.35, *P* = 0.03 (MBI)]. There was no significant difference in the occurrence of adverse events (AE) between acupuncture treatment combined with CR and CR alone (*P* > 0.05). The certainty of the evidence was rated low level because of flaws in the study design and considerable heterogeneity among the included studies.

**Conclusion:**

This review found that acupuncture treatment combined with CR may have a positive effect on improving cognitive function and self-care ability in PSCI patients. However, our findings should be treated with caution owing to the existence of methodological quality issues. High-quality studies are urgently required to validate our results in the future.

**Systematic review registration:**

https://www.crd.york.ac.uk/prospero/display_record.php?ID=CRD42022338905, identifier: CRD42022338905.

## 1. Introduction

Stroke is the second leading cause of adult disability and death around the world (1). It is well established that cognitive impairment is one of the major functional disorders after stroke. It is a clinical syndrome characterized by cognitive impairment that occurs after stroke events and persists for 3 to 6 months, mainly in the cognitive domains of language, computation, memory and executive skills (2). Post-stroke cognitive impairment (PSCI) is likely to be ignored because it is masked by severe physical disability. However, the prevalence of PSCI in stroke survivors is estimated to be 20–80%, depending on race, country, and diagnostic criteria (3). Although the prevalence of PSCI is high based on current data, there is still evidence that existing criteria may underestimate the incidence of cognitive decline in stroke survivors (4). PSCI is also associated with a decrease in the ability to care for themselves, which increases the burden on caregivers and reduces the patient's quality of life (5). At the same time, PSCI can place a serious financial strain on the patient's family. When cognitive decline occurs, the expenditures on care are three times higher than when there is no cognitive decline (6). Therefore, finding safe and effective therapies for PSCI is a major challenge for post-stroke rehabilitation.

PSCI is an important subtype of vascular dementia, which is preventable and treatable compared to dementia caused by neurodegenerative diseases such as Alzheimer's disease. At present, the main treatments for PSCI include drug therapy, repetitive transcranial magnetic stimulation (r TMS), and cognitive rehabilitation (CR) (7–9). However, these treatments have some shortcomings. Acetylcholinesterase inhibitors are recommended to improve cognitive function in stroke survivors. However, long-term intake of this medication can cause serious side effects such as gastrointestinal reactions, hepatotoxicity, and systemic symptoms such as insomnia and fatigue (10). R TMS is a form of non-invasive brain stimulation (NIBS). Most of the existing studies have focused on the effectiveness of r TMS in the treatment of post-stroke limb paralysis and dysphagia (11, 12). To date, there is no high-quality evidence that r TMS has a positive effect on the recovery of cognitive function in PSCI patients. Cognitive rehabilitation (CR) has a beneficial effect in the comprehensive treatment of stroke and can effectively promote the recovery of cognitive function in PSCI patients. However, CR also suffers from irreparable deficiencies. To achieve the desired therapeutic effect, cognitive rehabilitation is often conducted one-on-one between the rehabilitation trainer and the patient, requiring long-term cooperation from the patients. Meanwhile, due to differences in medical conditions, there can be significant differences in the skill level of rehabilitation trainers, which ultimately affects the therapeutic benefit of CR (13). In conclusion, there is still no officially approved pharmacological for the treatment of PSCI and treatment options are still limited (14).

As a basic therapy used in traditional Chinese medicine (TCM) for the prevention and treatment of diseases, acupuncture has been used clinically in China for thousands of years (15). Based on the application site, acupuncture can be divided into abdominal acupuncture (AA) and scalp acupuncture (SA). Furthermore, based on the operation method, acupuncture can be classified into manual acupuncture (MA) and electro-acupuncture (EA), which have proven to have the advantages of low price, excellent effect and simple operation (16). In addition, as a non-pharmacological intervention, acupuncture has better efficacy for chronic conditions that are difficult to treat with traditional treatment modalities, such as low back pain and kidney diseases (17, 18). As a result of these advantages, acupuncture has received continued interest from the general public and health professionals (19). Furthermore, a growing number of healthcare institutions have used acupuncture clinically and have shown that acupuncture can be a prospective therapeutic measure to enhance cognitive function in patients with PSCI (20, 21). Meanwhile, animal models of PSCI have been used to explore the potential mechanisms of acupuncture treatment to achieve maximum benefit (22, 23).

In the past years, three reviews regarding acupuncture treatment for PSCI have been published. The meta-analysis published in 2017 (24) included only 11 studies with a total of 789 patients, which suffers from an inadequate sample size. Meanwhile, the study only assessed the effect of electro-acupuncture on cognitive function in patients with PSCI, and electro-acupuncture only represents one type of acupuncture, making the findings inevitably limited and failing to provide convincing support for clinical application. Two meta-analyses (25, 26), published in 2020 and 2021 respectively, selected the Mental State Examination Scale (MMSE) and the Montreal Cognitive Assessment Scale (MoCA) as outcome indicators. The findings revealed that acupuncture treatment was able to achieve higher scores in terms of cognitive function improvement compared to sham acupuncture or pharmacotherapy. However, neither study examined whether PSCI patients' ability to care for themselves improved after receiving acupuncture treatment. Given the limitations of these previous reviews, the clinical conclusion to data have shown that there is insufficient evidence to support the routine use of acupuncture to enhance cognitive function and self-care ability in PSCI patients, with particular attention to the latter appearing extremely inadequate. Furthermore, with the widespread availability of acupuncture treatment, an increasing number of studies have been published in recent years (27, 28). Therefore, the aim of this review is to investigate the effects of acupuncture treatment on cognitive function and self-care ability in PSCI patients and to update the previously published reviews.

## 2. Materials and methods

### 2.1. Protocol and registration

The detailed protocol of this systematic review and meta-analysis has been registered on the international systematic review registration platform (PROSPERO) with the registration number is CRD42022338905 (29).

### 2.2. Search strategy

Randomized controlled trials (RCTs) regarding the acupuncture for the treatment of PSCI published from inception until May 2022 were searched in eight databases including Web of Science, Embase, PubMed, Cochrane Central Register of Controlled Trials, China Biomedical Literature Database (CBM), China Science and Technology Journal (VIP) database, the China National Knowledge Infrastructure (CNKI) database, and Wan fang database. Meanwhile, the reference lists of articles identified for inclusion were screened to identify as many relevant articles as possible. No language restrictions were used in the search process. The search terms included “acupuncture treatment,” “electro-acupuncture,” “cognitive impairment,” “PSCI,” “stroke,” “cerebrovascular accident,” “Zhen jiu liao fa” and “Hao Zhen.” The specific search strategies are described in the Supplementary Appendix.

### 2.3. Literature selection criteria

Two researchers independently screened and checked the titles and abstracts of the literature to be initially included based on the PICOS principles (patient, intervention, control, outcome, and study). The PICOS criteria used for literature screening were detailed as follows: (a) Types of participants: (1) Patients were diagnosed with a stroke, with no restrictions on their age, gender, and the duration of disease. (2) Their condition was verified by magnetic resonance imaging (MRI) or electronic computed tomography (CT). (3) Cognitive impairment was caused by stroke and not by other diseases, such as diabetes, cranial trauma, and Alzheimer's disease (AD); (b) Type of intervention: The experimental group was treated with manual acupuncture (MA) or electro-acupuncture (EA), combined or not with the same therapies as the control group. For the control group, cognitive rehabilitation (CR) must be used and all other types of interventions should be excluded; (c) Types of outcomes: (1) The primary outcome indicators focused on changes in cognitive function and could be assessed by the Montreal Cognitive Assessment Scale (MoCA), the Mental State Examination Scale (MMSE), and the Loewenstein Occupational Therapy Cognitive Assessment (LOTCA); (2) The secondary outcome indicators were used to evaluate the patient's ability to conduct activities of daily living and include the Modified Barthel Index (MBI) and Functional Independence Measure (FIM); (d) Types of study: Randomized Controlled Trials (RCTs). The safety of acupuncture therapy was evaluated by the severity and number of adverse events (AEs). The language type of the literature was limited to Chinese or English.

Meanwhile, we excluded literature that met the following criteria: (1) Duplication of publications; (2) Studies compared different acupuncture therapies; (3) Full text is not available; (4) Lack of effective outcome measures; (5) The types of studies are reviews, animal experiments, conference articles, and case reports.

### 2.4. Data collection process

Two researchers independently used pre-designed forms to retrieve useful information from qualified studies, including publication year, first author, sample size, mean age and method used in experimental groups (e.g., acupuncture modality and acupoint selection), and duration and frequency of treatment. Any inconsistencies in information extraction could be resolved by consulting Chen FY. After data extraction was completed, researchers assessed the safety of acupuncture treatment by collecting adverse event reports from the included articles.

### 2.5. Study quality assessment

Two researchers independently assessed the methodological quality of the included RCTs according to the criteria detailed in Cochrane's Risk of Bias (ROB) tool (30). Any inconsistencies related to the assessment results were resolved by Chen FY. The Cochrane's ROB assessment tool consists of seven components, which are random sequence generation, allocation concealment, blinding of participants and personnel, blinding of outcome assessors, incomplete outcome data, selective reporting and other biases. Each component can be classified as one of the following levels: “low risk of bias,” “uncertain risk of bias” and “high risk of bias.”

### 2.6. GRADE assessment

Two researchers (Y-L and XP-L) assessed the quality of evidence for each outcome indicator by using the Grading of Recommendations, Assessment, Development, and Evaluation (GRADE) system (31). When disagreements arose, they could be resolved through consultation or by consulting a third researcher. The quality of evidence for each outcome is displayed in the form of a GRADE evidence profile to determine the certainty of all pooled outcomes. The GRADE system includes five downgrading factors and three escalating factors, five downgrading factors consisting of risk of bias, inconsistency, indirectness, uncertainty, and publication bias, and three escalating factors consisting of larger effect values, dose-effect relationships, and negative bias. The quality of evidence for each outcome was assessed by the eight factors mentioned above, which ultimately resulted in a high, moderate, low, or very low evidence level.

### 2.7. Statistical analysis

After data extraction was completed, Rev Man 5.4 software (Cochrane Collaboration, Oxford, United Kingdom) was selected for statistical analysis. If the outcome indicator was a continuous variable, mean difference (MD) or standardized mean difference (SMD) and 95% confidence interval (CI) were selected for calculation. If the outcome indicator was a dichotomous variable, the risk ratio (RR) and 95% CI were selected for calculation. Heterogeneity between included studies was assessed using Q-tests (*P*-values) and the I^2^ statistic, and effect models were selected accordingly. If *P* < 0.1 and I^2^ > 50%, statistically significant heterogeneity among the included studies was detected and a random effects model was selected to calculate the effect size. Conversely, if *P* ≥ 0.1 and I^2^ ≤ 50%, the heterogeneity among the included studies was regarded as tolerable and we selected the fixed effects model to merge the data. This meta-analysis assessed the significance of the pooled results by Z-test, with *P* < 0.05 being a statistically significant difference.

We categorized the included studies according to different acupuncture types and treatment duration, which were MA, EA, 0–4 weeks, and >4 weeks. Subgroup analysis was attempted to account for possible heterogeneity under the stratification factors of different acupuncture types and treatment cycles. Sensitivity analyses were used to validate the robustness of the meta-analysis results and to explore potential sources of heterogeneity by excluding each individual study in the original analysis. For cognitive function and self-care ability, we used funnel plots and Egger's tests to evaluate publication bias in the included studies.

## 3. Results

### 3.1. Description of the studies

**Figure 1:**
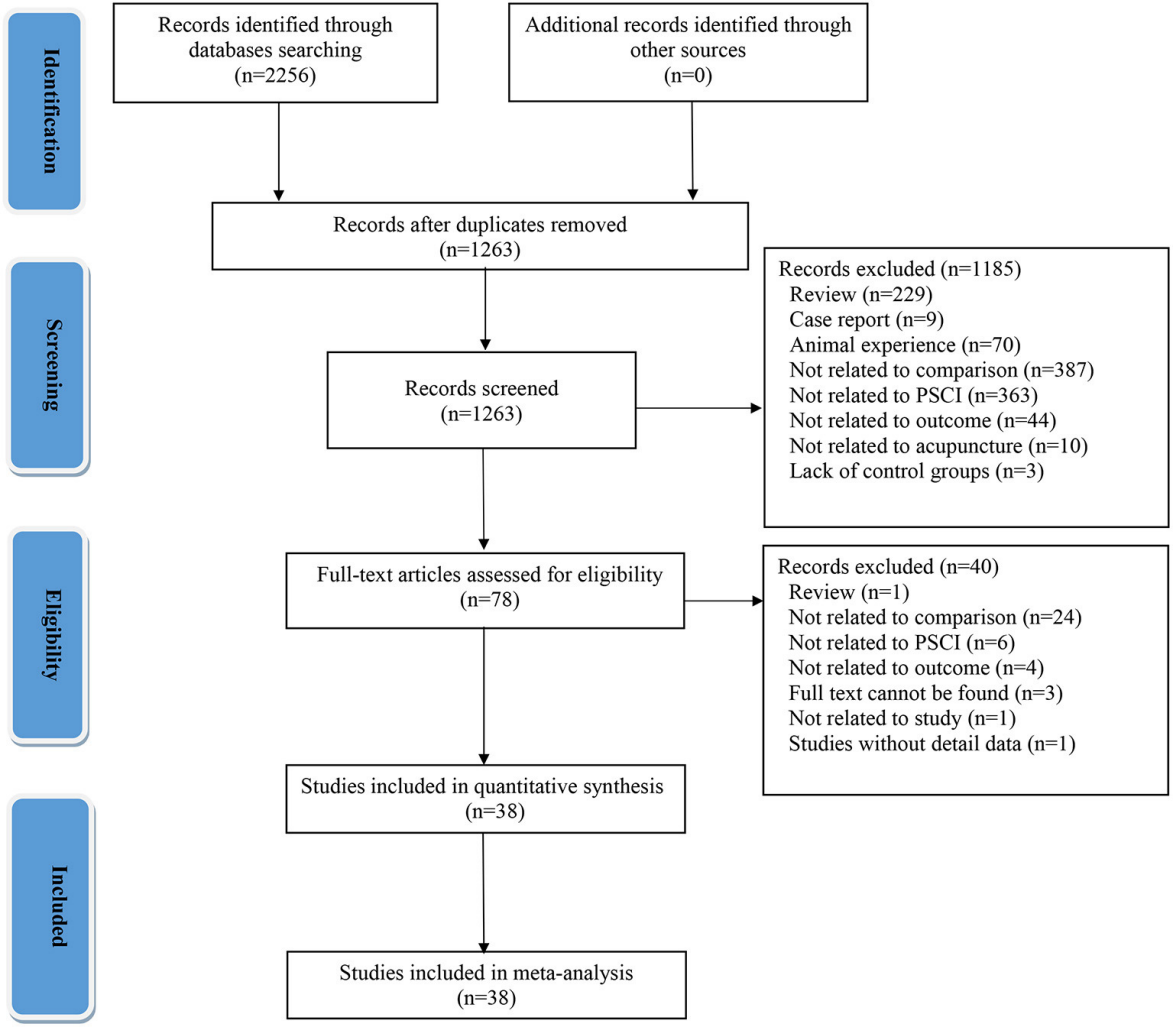
Flow chart of the literature screening process.

Using a pre-defined search strategy, we initially retrieved 2,256 relevant records from eight databases. A total of 2,178 duplicate and irrelevant studies were excluded by reading the titles and abstracts of the articles. Then, the full text of the remaining studies was read and 40 records were excluded. Finally, 38 studies were included in the qualitative analysis (32–69). All included 38 studies conducted in China, of which 36 and 2 RCTs were published in Chinese and English, respectively. These included studies were reported between 2013 and 2022. A flow chart of the literature screening process for the systematic review and meta-analysis is shown in Figure 1.

### 3.2. Description of participants

A total of 2,971 participants were included, of which 1,486 participants were in the experimental group and 1,485 participants were in the control group. The sample size ranged from 40 to 150. The proportion of males was higher than females. The mean age of patients was similar in both groups but was not reported in four studies (34, 45, 49, 59). Eight studies (38, 48–50, 58, 59, 68, 69) reported on patient dropouts and provided corresponding reasons, ranging from 2 to 17 individuals. Details of the 38 included RCTs are summarized in Table 1.

**Table 1:** Characteristics of the included studies.

**References**	**Sample size** **(EG/CG)**	**Man (%)**	**Mean age** **(EG/CG)**	**Type of stroke**	**Disease duration (EG/CG)**	**Method of intervention**	**Comparison**	**Outcome**	**Loss situation** **(EG/CG)**
Teng et al. (32)	55/55	65	59.47 ± 8.62/60.17 ± 10.68	Ischemic stroke or hemorrhage	60.17 ± 10.68/59.67 ± 11.28 d	MA+CR	CR	①③	N
Mao (33)	39/39	47	57.3 ± 15.6/57.3 ± 15.6	Ischemic stroke or hemorrhage	78.2 ± 38.2/76.3 ± 37.3 d	MA+CR	CR	②④	N
Liu (34)	25/25	68	/	Ischemic stroke or hemorrhage	/	EA+CR	CR	①	N
Wang et al. (35)	30/30	65	53.27 ± 11.62/56.73 ± 9.32	Ischemic stroke or hemorrhage	/	EA+CR	CR	①④	N
Niu et al. (36)	75/75	54	52.06 ± 7.98/51.89 ± 10.24	Ischemic stroke	/	MA+CR	CR	①②④	N
Liao et al. (37)	30/30	55	56.2 ± 6.5/56.6 ± 6.6	Ischemic stroke or hemorrhage	54.6 ± 6.3/53.9 ± 6.5 d	MA+ CR	CR	③④	N
Yang et al. (38)	34/33	55	68.00 ± 8/67.00 ± 8	/	14.7 ± 6.2/15.5 ± 6.2 d	MA+CR	CR	①②	2/1
Wen et al. (39)	30/30	52	64.00 ± 7.18/63.7 ± 6.98	Ischemic stroke or hemorrhage	5.70 ± 1.92/5.3 ± 1.90 m	MA+CR	CR	①④	N
Jiang et al. (40)	40/40	58	60.15 ± 6.32/58.67 ± 8.03	Ischemic stroke	10.01 ± 3.29/9.85 ± 3.01 d	MA+CR	CR	③④	N
Hu (41)	63/63	48	70.03 ± 2.29/69.96 ± 2.20	Ischemic stroke or hemorrhage	2.45 ± 0.34/2.56 ± 0.31 m	MA+CR	CR	②	N
Zhang et al. (42)	30/30	63	70.10 ± 4.51/69.03 ± 4.70	Ischemic stroke	23.03 ± 7.47/24.63 ± 11.77 d	EA+CR	CR	②	N
Wang (43)	20/20	30	61.8 ± 11.92/60.25 ± 10.39	Ischemic stroke or hemorrhage	30.20 ± 11.83/28.25 ± 13.01 d	MA+CR	CR	③④	N
Wang et al. (44)	40/40	61	45.39 ± 11.42/42.29 ± 11.72	Ischemic stroke	24.05 ± 11.89/26.85 ± 16.10 d	MA+CR	CR	①	N
Liu (45)	56/56	55	/	Ischemic stroke	/	MA+CR	CR	①②	N
Xie et al. (46)	47/47	53	55.16 ± 1.23/55.43 ± 1.17	Ischemic stroke	89.36 ± 11.45/90.72 ± 11.64 d	MA+CR	CR	①②	N
Yang (47)	40/40	58	51.35 ± 7.30/51.27 ± 7.46	/	21.40 ± 5.38/21.57 ± 5.54	MA+CR	CR	①④	N
Zhuo et al. (48)	20/22	55	63.25 ± 9.34/63.04 ± 9.16	Ischemic stroke	2.15 ± 1.03/2.27 ± 1.06 m	MA+CR	CR	②	3/2
Yang (49)	47/47	55	/	Ischemic stroke or hemorrhage	/	MA+CR	CR	①④	0/3
Yao et al. (50)	40/38	53	61.27 ± 5.38/62.27 ± 6.48	/	42.38 ± 14.23/44.23 ± 18.87 d	EA+CR	CR	②⑤	5/7
Wang et al. (51)	50/50	50	53.8 ± 11.7/54.5 ± 13.6	Ischemic stroke or hemorrhage	/	MA+CR	CR	①②④	N
Xing et al. (52)	30/26	57	63.4 ± 8.5/60.9 ± 8.1	Ischemic stroke or hemorrhage	/	MA+CR	CR	③④	N
Piao (53)	53/53	63	60.37 ± 5.45/59.15 ± 5.29	Ischemic stroke	5.12 ± 1.39/5.33 ± 1.40 d	MA+CR	CR	①	N
Qian et al. (54)	35/35	53	70 ± 6/69 ± 6	Ischemic stroke	10.14 ± 3.37/10.54 ± 3.85 d	MA+CR	CR	①	N
Wang et al. (55)	40/40	63	66 ± 8/67 ± 9	Ischemic stroke	14.62 ± 6.17/15.45 ± 6.24 d	MA+CR	CR	①②④	N
Song et al. (56)	35/35	57	60 ± 10/58 ± 10	Ischemic stroke or hemorrhage	2.8 ± 1.4/2.5 ± 1.6 m	MA+ CR	CR	②④	N
Lin et al. (57)	30/29	63	65 ± 5/67 ± 7	Ischemic stroke	29.85 ± 18.10/30.05 ± 19.89 d	EA+CR	CR	②	N
Zhang et al. (58)	28/30	52	59.95 ± 8.71/61.12 ± 9.62	Ischemic stroke or hemorrhage	39.72 ± 18.73/42.11 ± 17.56 d	MA+CR	CR	①④	7/5
Zeng et al. (59)	34/35	58	/	Ischemic stroke or hemorrhage	71.00 ± 42.76/68.00 ± 36.56 d	MA+CR	CR	①④	6/5
Tian et al. (60)	25/25	58	68.19 ± 0.16/68.21 ± 0.15	Ischemic stroke or hemorrhage	/	MA+CR	CR	①	N
Li (61)	60/60	61	60.25 ± 6.34/60.26 ± 6.33	Ischemic stroke	/	MA+CR	CR	①	N
Wei et al. (62)	30/30	62	60.32 ± 7.93 ± 60.38 ± 8.01	Ischemic stroke	/	EA+CR	CR	③	N
Zhang et al. (63)	50/50	49	63.5 ± 3.4/64.1 ± 3.6	Ischemic stroke	/	MA+CR	CR	①⑤	N
Zhou (64)	30/30	63	53.76 ± 9.27/53.89 ± 9.52	Ischemic stroke	64.35 ± 31.65/64.15 ± 30.97 d	MA+CR	CR	①②	N
Chen (65)	48/48	53	60.47 ± 2.35/60.80 ± 2.19	Ischemic stroke or hemorrhage	42.66 ± 7.89/42.50 ± 7.31 d	MA+CR	CR	①④	N
Du (66)	30/30	48	62.14 ± 9.48/66.49 ± 10.03	Ischemic stroke	26.00 ± 0.01/25.00 ± 0.01 d	MA+CR	CR	②④	N
Chen et al. (67)	30/30	45	62.00 ± 5.12/61.77 ± 4.81	Ischemic stroke or hemorrhage	42.00 ± 5.52/43.40 ± 5.10 d	MA+CR	CR	①③	N
Xiong et al. (68)	35/35	53	63.0 ± 7.23/65.3 ± 8.52	Ischemic stroke or hemorrhage	2.13 ± 0.85/2.51 ± 0.46 m	MA+CR	CR	①③	1/1
Jiang et al. (69)	52/51	47	62.33 ± 7.72/62.37 ± 7.89	Ischemic stroke or hemorrhage	41.13 ± 18.80/44.22 ± 17.00 d	MA+CR	CR	①④⑤	9/8

EG, Experimental group; CG, Control group; MA, manual acupuncture; EA, electroacupuncture; CR, cognitive rehabilitation; ① MMSE, Mental State Examination Scale; ② MoCA, Montreal Cognitive Assessment Scale; ③ LOTCA, Loewenstein Occupational Therapy Cognitive Assessment; ④ MBI, Modified Barthel Index; ⑤ FIM, Functional Independence Measure; N, no loss situation; /, not mention.

### 3.3. Description of interventions

In the included studies, scalp acupuncture and manual acupuncture were used with equal frequency (42.1%), followed by electroacupuncture (15.8%). All included studies used cognitive rehabilitation (CR) as a control measure. The duration of needle retention was set between 5 min to 24 h, with the majority of studies set at 30 min (*n* = 25). The treatment frequency ranged from 3 to 7 times per week, with a popular pattern of 6 times per week (*n* = 15). The studies varied widely in the treatment period, ranging from 3 to 12 weeks, with 8 weeks treatment period as the most preferred (*n* = 16). By observing the pattern of acupoint selection in the included studies, we found that acupoints selected for acupuncture treatment of PSCI were mainly located in the cephalic region. Bai-hui (81.6%), Si-shen-cong (52.6%), Shen-ting (36.8%), Nei-guan (18.4%), Feng-chi (15.8%), Shui-gou (15.8%), Qu-bin (13.2%), and Xuan-li (13.2%) were the acupoints used most frequently. Table 2 elaborates on the characteristics of the interventions in the included literature.

**Table 2:** Details of interventions in included studies.

**References**	**Intervention methods**		**Treatment duration**			**Adverse events**
	**Style**	**Acupoint selection**	**Frequency (t/w)**	**Retained (min/h)**	**Course (w)**	
Teng et al. (32)	MA	The upper-jiao, Lower-jiao, Liver, Kidney, and heart regions in the eye's area	7 times/w	5 min	4 weeks	/
Mao (33)	SA	Bai-hui (DU20), Shen-ting (DU24), Qian-ding (DU21), Xuan-li (GB6), Qu-bin (GB7), Mei-chong (BL3), Tou-lin-qi (GB15)	5 times/w	30 min	6 weeks	/
Liu (34)	EA	Shen-ting (DU24), Bai-hui (DU20)	6 times/w	60 min	4 weeks	/
Wang et al. (35)	EA	Bai-hui (DU20), Zu-san-li (ST36)	6 times/w	30 min	8 weeks	/
Niu et al. (36)	SA	Bai-hui (DU20), Qian-ding (DU21), Lu-hui (DU22), Qian ting (DU21)	6 times/w	6 h	8 weeks	/
Liao et al. (37)	MA	The upper-jiao, Kidney, and spleen regions in the eye's area	5 times/w	5 min	8 weeks	/
Yang et al. (38)	SA	Bai-hui (DU20)	3 times/w	24 h	4 weeks	Subcutaneous edema and needle fainting
Wen et al. (39)	MA	Shui-gou (DU26), Yin-tang (EX-HN3), Shen-ting (DU24), Bai-hui (DU20), Ya-men (DU15), Feng-fu (DU16), Yao-yang-guan (DU3), Ming-men (DU4)	7 times/w	30 min	6 weeks	/
Jiang et al. (40)	MA	The upper-jiao and lower-jiao regions in the eye's area	7 times/w	20 min	8 weeks	/
Hu (41)	MA	Bai-hui (DU20), Ming-men (DU4), Si-shen-cong (EX-HN1), Yao-yang-guan (DU3), Tai-xi (KI3), Shen-shu (BL23), Feng-shi (GB20)	5 times/w	30 min	8 weeks	/
Zhang et al. (42)	EA	Shen-ting (DU24), Qian-ding (EX-HN1), Xuan li (GB6)	5 times/w	30 min	6 weeks	/
Wang (43)	SA	Qian-ding (EX-HN1), Xuan li (GB6), Bai-hui (DU20), Qu-bin (GB7)	6 times/w	30 min	8 weeks	/
Wang et al. (44)	SA	Bai-hui (DU20), Qian-ding (EX-HN1), Lu-hui (DU22), Shen-ting (DU24)	6 times/w	6~8 h	6 weeks	/
Liu (45)	SA	Bai-hui (DU20)	3 times/w	24 h	4 weeks	/
Xie et al. (46)	MA	Bai-hui (DU20), Yin-tang (EX-HN3), Shui-gou (DU26), Nei-guan (PC6)	3 times/w	30 min	4 weeks	/
Yang (47)	MA	Shen-shu (BL23), Feng-fu (DU16), Bai-hui (DU20), Yong-quan (KI1), Xuan-zhong (GB39)	6 times/w	30 min	4 weeks	/
Zhou et al. (48)	MA	Da-ling (PC7), Nei-guan (PC6), Shen-men (HT7), Tong-li (HT5), Shen-ting (DU24), Qian-ding (EX-HN1), Lu-hui (DU22), Hou-ding (DU19), Bai-hui (DU20), Jiao-sun (SJ20), Shuai-gu (GB8)	5 times/w	30 min	3 weeks	/
Yang (49)	SA	Bai-hui (DU20), Si-shen-cong (EX-HN1), Shen-men (HT7), Hou-xi (SI3), Zhao-hai (KI6), Xuan-zhong (GB39)	6 times/w	30 min	4 weeks	/
Yao et al. (50)	EA	Bai-hui (DU20), Shen-ting (DU24), Ben-shen (BG13)	5 times/w	30 min	12 weeks	Subcutaneous edema
Wang et al. (51)	SA	Bai-hui (DU20), Qu-bin (GB7), Qian-ding (EX-HN1), Xuan li (GB6)	6 times/w	6 h	3 weeks	/
Xing et al. (52)	SA	Bai-hui (DU20), Qu-bin (GB7), Qian-ding (EX-HN1), Xuan li (GB6)	6 times/w	6 h	8 weeks	/
Piao (53)	MA	Bai-hui (DU20), Si-shen-cong (EX-HN1), San-yin-jiao (SP6), Wei-zhong (BL40), Shui-gou (DU26), He-gu (LI4), Nei-guan (PC6)	7 times/w	30 min	12 weeks	/
Qian et al. (54)	SA	Bai-hui (DU20)	3 times/w	24 h	8 weeks	/
Wang et al. (55)	MA	Bai-hui (DU20), Shen-ting (DU24)	3 times/w	30 min	8 weeks	/
	**Style**	**Acupoint selection**	**Frequency (t/w)**	**Retained (min/h)**	**Course (w)**	
Song et al. (56)	MA	Bai-hui (DU20), Ming-men (DU4), Si-shen-cong (EX-HN1), Yao-yang-guan (DU3), Tai-xi (KI3), Shen-shu (BL23), Feng-shi (GB20)	5 times/w	30 min	8 weeks	/
Lin et al. (57)	EA	Bai-hui (DU20)	6 times/w	20 min	4 weeks	/
Zhang et al. (58)	MA	Dan-zhong (RN17), Zhong-wan (RN12), Qi-hai (RN6), Xue-hai (SP10), Zu-san-li (ST36), Wai-guan (SJ5)	6 times/w	30 min	8 weeks	/
Zeng et al. (59)	SA	Shen-ting (DU24), Si-shen-cong (EX-HN1), Ben-shen (BG13), Nao-hu (DU17), Nao-kong (GB19)	6 times/w	30 min	4 weeks	/
Tian et al. (60)	MA	Bai-hui (DU20), Si-shen-cong (EX-HN1), Feng-chi (GB20), Qu-chi (LI11), Shen-ting (DU24), Nei-guan (PC6), Wai-guan (SJ5), Xue-hai (SP10)	7 times/w	30 min	8 weeks	/
Li (61)	SA	Bai-hui (DU20), Si-shen-cong (EX-HN1), Shui-gou (DU26), Nei-guan (PC6)	5 times/w	30 min	12 weeks	/
Wei et al. (62)	EA	Bai-hui (DU20), Qian-ding (EX-HN1), Shen-ting (DU24), Qu-bin (GB7)	5 times/w	30 min	6 weeks	/
Zhang (63)	MA	Bai-hui (DU20), Si-shen-cong (EX-HN1), Shui-gou (DU26), Yin-tang (EX-HN3), Zu-san-li (ST36), Fe-long (ST40)	6 times/w	30 min	8 weeks	/
Zhou (64)	MA	Bai-hui (DU20), Yin-tang (EX-HN3), Si-shen-cong (EX-HN1), Nei-guan (PC6), Shui-gou (DU26)	3 times/w	30 min	8 weeks	/
Chen (65)	MA	Bai-hui (DU20), Nei-guan (PC6), Zu-san-li (ST36), Shen-ting (DU24), Wai-guan (SJ5), Qu-chi (LI11), Si-shen-cong (EX-HN1), Feng-chi (GB20), Xue-hai (SP10), Feng-shi (GB31), Yang-lin-quan (GB34)	7 times/w	30 min	4 weeks	/
Du (66)	SA	Si-shen-cong (EX-HN1), Ben-shen (BG13), Lu-hui (DU22), Feng-chi (GB20)	5 times/w	30 min	8 weeks	/
Chen et al. (67)	SA	Bai-hui (DU20), Si-shen-cong (EX-HN1)	6 times/w	30 min	4 weeks	/
Xiong et al. (68)	SA	Bai-hui (DU20), Si-shen-cong (EX-HN1), Feng-chi (GB20), Shen-ting (DU24)	6 times/w	3~4 h	8 weeks	/
Jiang et al. (69)	SA	Bai-hui (DU20), Shen-ting (DU24)	5 times/w	30 min	12 weeks	N

MA, manual acupuncture; EA, electro-acupuncture; SA, scalp acupuncture; N, no adverse events; /, not mention.

### 3.4. Methodological quality

**Figure 2:**
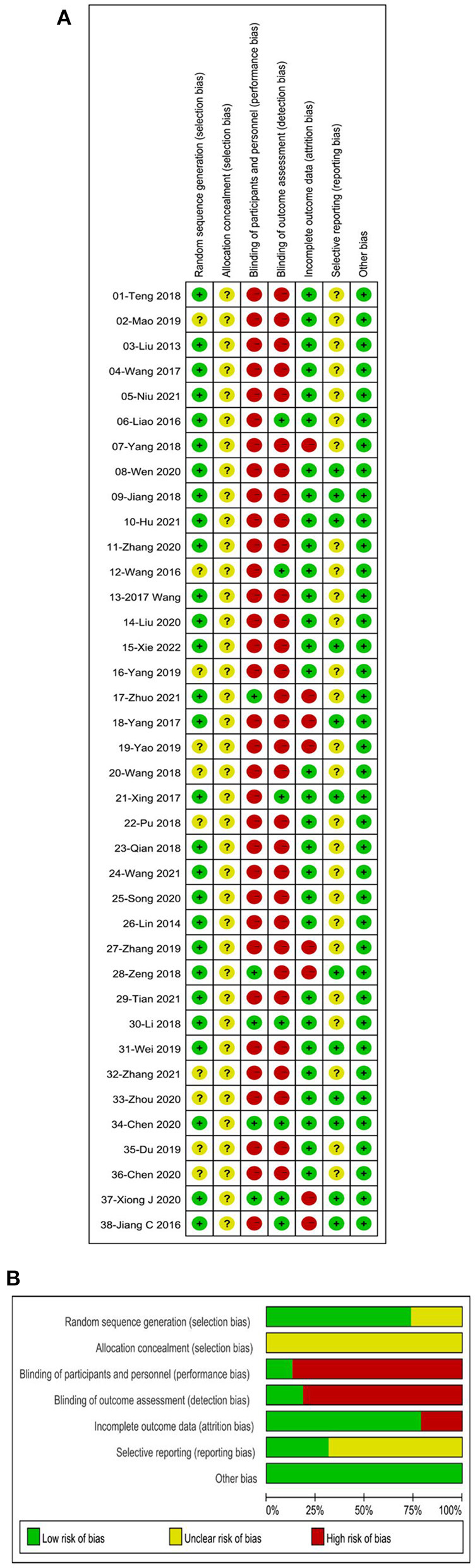
Risk of bias (ROB) assessments of included studies. **(A)** ROB graph. **(B)** ROB summary.

The methodological quality of the included studies was universally unsatisfactory. Twenty-eight studies (32, 34–42, 44–46, 48, 49, 52, 54–62, 65, 68, 69) (73.7%) reported their sequence generation methods by random number tables or computer randomization groups, while the other studies (33, 43, 47, 50, 51, 53, 63, 64, 66, 67) (26.3%) simply mentioned the word “randomization” and were therefore rated as “unclear risk.” Details of allocation concealment were not reported in the included literature. Methods regarding blinding were not reported in more than 75% of the studies, with three studies (61, 65, 68) (8%) reported double blinding, four studies (37, 43, 52, 69) (11%) reported blinding for outcome assessors, and two studies (48, 59) (5%) specified the blinding of operators. The eight studies (38, 48–50, 58, 59, 68, 69) (21.1%) were rated as high risk because they did not conduct appropriate intention-to-treat analysis in the context of the dropout situation. In terms of selective reporting, twelve studies (39–41, 46, 49, 52, 59, 62, 64–66, 68) (31.5%) submitted protocols to the ethics committee and received approval, while the remaining studies were rated as “unclear risk.” Other bias was not found in the included studies. The results of the ROB assessment are illustrated in Figure 2.

### 3.5. Meta-analysis

#### 3.5.1. Global cognitive function

##### 3.5.1.1. Mental state examination scale

**Figure 3:**
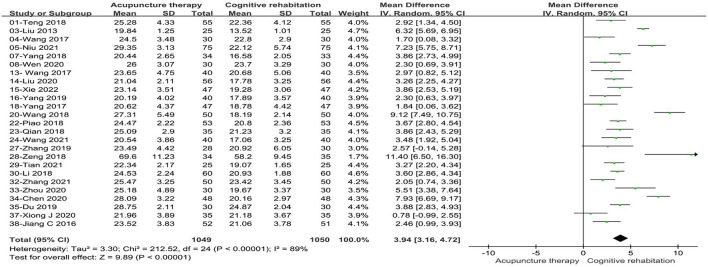
Forest plot of acupuncture treatment combined with CR vs. CR on MMSE scale.

Twenty-five studies (32, 34–36, 38, 39, 44–47, 49, 51, 53–55, 58–61, 63–66, 68, 69) involving 2099 patients reported an increase in MMSE scores with acupuncture treatment combined with CR compared to CR alone. These studies showed statistical heterogeneity in MMSE scale (*P* < 0.00001, I^2^ = 89%). The merged MD value using a random effects model was 3.94 (95%CI: 3.16–4.72). The results demonstrated that there was a statistically significant difference between the acupuncture treatment combined with CR and CR alone based on the total effect (Z = 9.89, *P* < 0.00001, see Figure 3).

**Figure 4:**
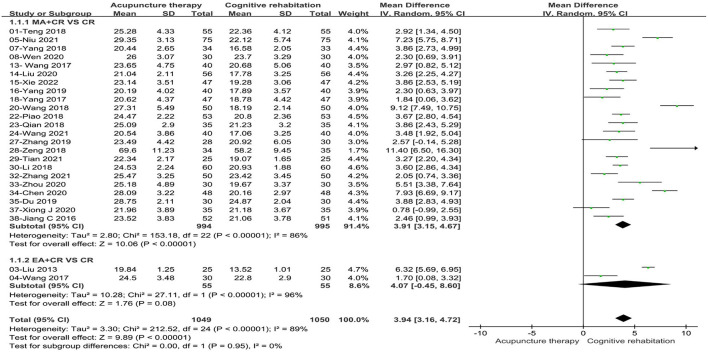
Forest plot of the intervention subgroup of acupuncture treatment combined with CR vs. CR on MMSE scale. MA, manual acupuncture; EA, electro-acupuncture; CR, cognitive rehabilitation.

**Figure 5:**
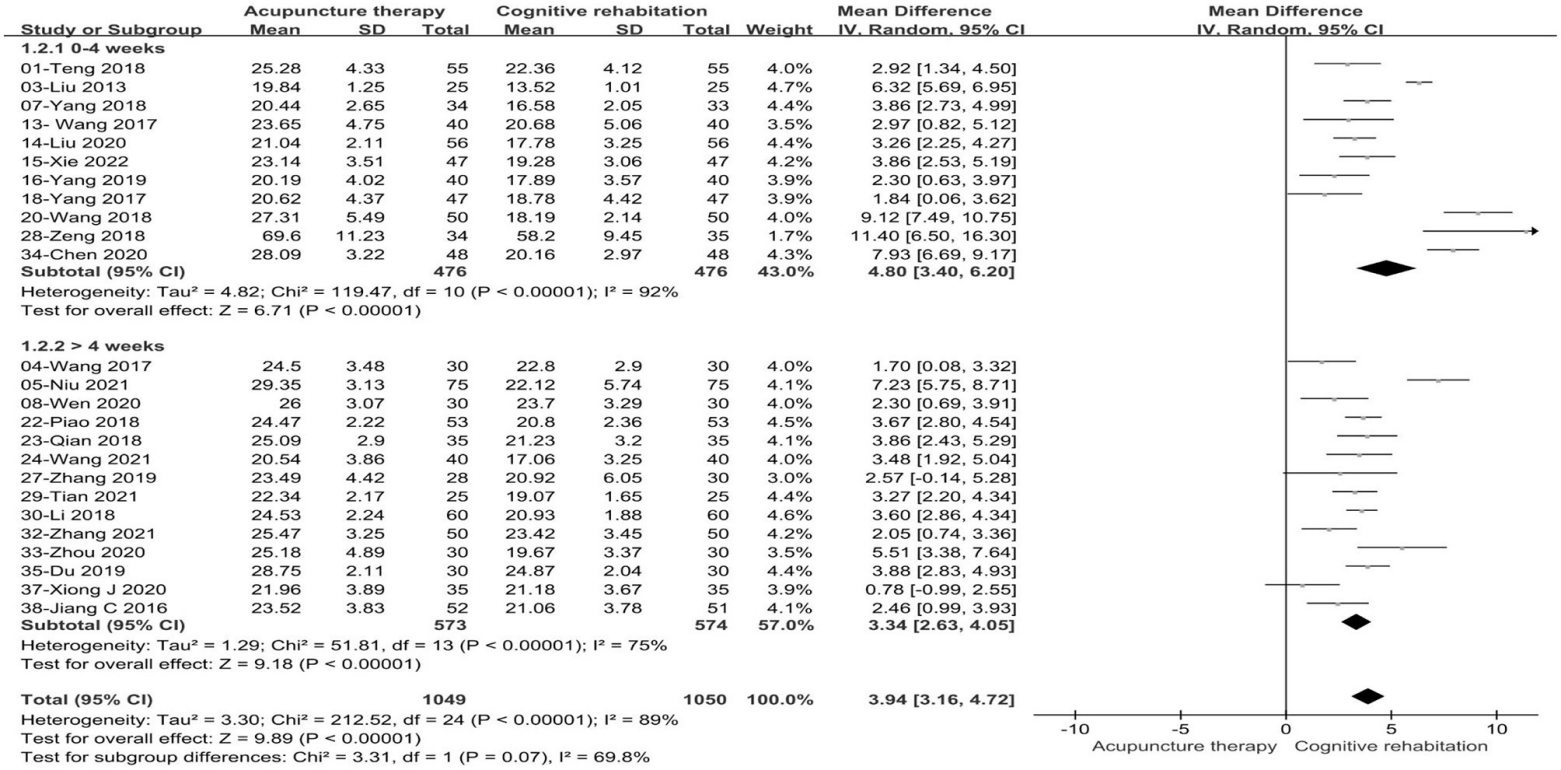
Forest plot of the treatment duration subgroup of acupuncture treatment combined with CR vs. CR on MMSE scale.

Considering the effect of acupuncture types and treatment duration on the merged data, we performed subgroup analysis on this basis. Subgroup based on the acupuncture types: we divided the types of acupuncture into manual acupuncture (MA) and electro-acupuncture (EA). Differences between subgroups were not statistically significant (*P* = 0.95, I^2^ = 0%). Two studies (34, 35) (8%) with 110 patients compared EA plus CR with CR and showed no improvement in cognitive function (MD: 4.07, 95%CI: −0.45–8.60, *P* = 0.08, see Figure 4). Meanwhile, twenty-three studies (32, 36, 38, 39, 44–47, 49, 51, 53–55, 58–61, 63–66, 68, 69) (92%) with 1,989 patients compared MA plus CR with CR and the combined results indicated that significant improvement in cognitive function compared to CR alone (MD:3.91, 95%CI:3.15–4.67, *P* < 0.00001, see Figure 4). Subgroup based on the treatment duration: we classified the included studies into two groups (0–4 weeks and > 4 weeks). Statistical differences were found between subgroups (*P* = 0.07, I^2^ = 69.8%). Subgroup analysis showed that both 0–4weeks (MD: 4.80, 95%CI: 3.40–6.20, *P* < 0.00001) and more than 4 weeks (MD: 3.34, 95%CI: 2.63–4.05, *P* < 0.00001) treatment duration were superior to the control group in terms of improving cognitive function (see Figure 5). Sensitivity analysis was then performed by removing the study with the maximum weight. Also, one study was excluded at a time while synthesizing the remaining RCTs to determine which study was the source of heterogeneity and to assess whether the merged results would be altered by the exclusion of a single study. In the sensitivity analysis, one study was identified as a possible source of heterogeneity in the >4 weeks subgroup. After excluding this study, the results of the meta-analysis remained stable and the heterogeneity was lower than before (see Supplementary Table 1). The retention time of needles was relatively lengthy in Niu's study (36) compared to the other studies, so it is speculated that this may have contributed to the heterogeneity. In the results of the sensitivity analysis for the acupuncture types subgroup, the exclusion of any of the studies had little effect on the MD values and failed to identify the source of heterogeneity.

##### 3.5.1.2. Montreal cognitive assessment scale

**Figure 6:**
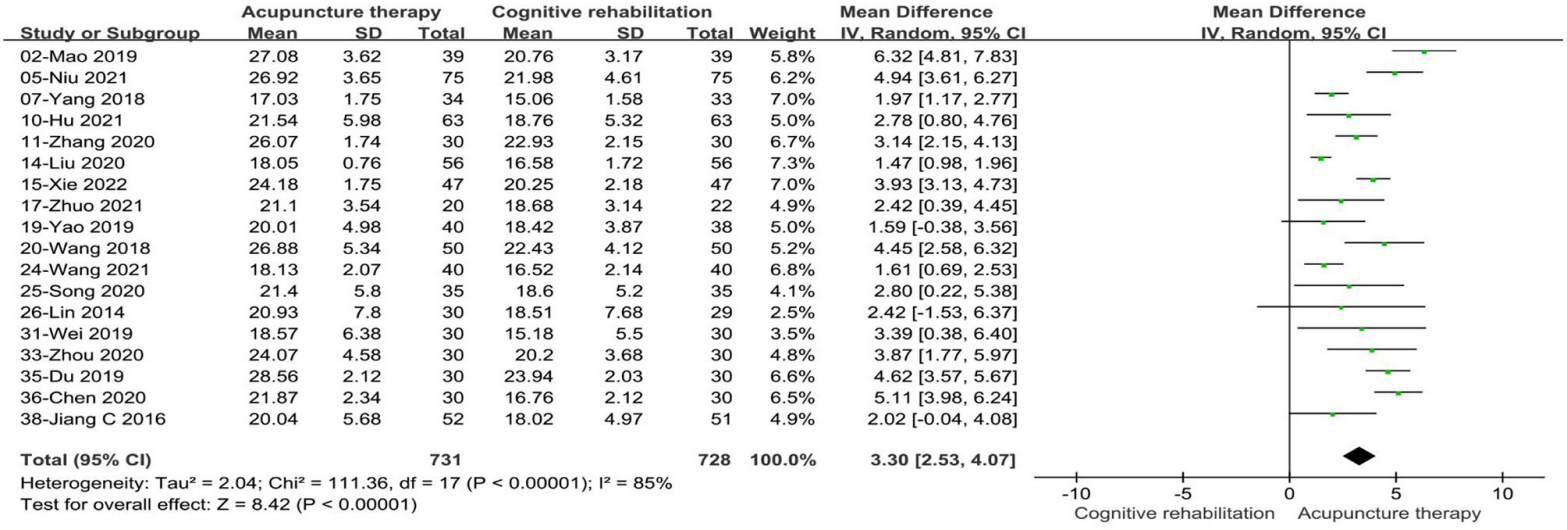
Forest plot of acupuncture treatment combined with CR vs. CR on MoCA scale.

Eighteen studies (33, 36, 38, 41, 42, 45, 46, 48, 50, 51, 55–57, 62, 64, 66, 67, 69) involving 1,459 patients reported a better performance in MoCA scores with acupuncture treatment combined with CR compared to CR alone. These studies displayed statistical heterogeneity in the MoCA scale (*P* < 0.00001, I^2^ = 85%). The pooled MD value using a random effects model was 3.30, (95%CI: 2.53–4.07). The results showed that there was a statistically significant difference between acupuncture treatment combined with CR and CR alone according to the measure of the total effect (Z = 8.42, *P* < 0.00001, see Figure 6).

**Figure 7:**
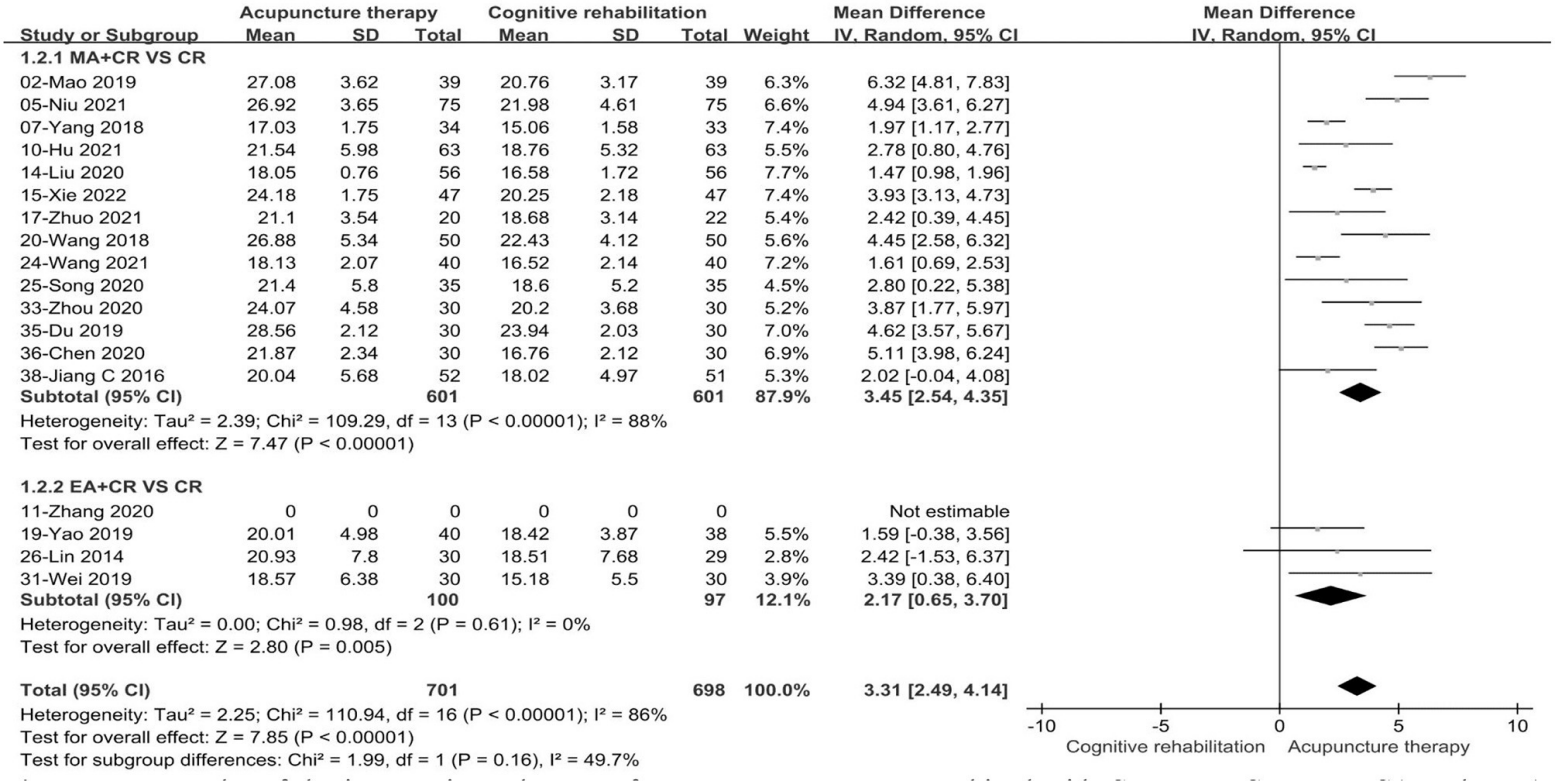
Forest plot of the intervention subgroup of acupuncture treatment combined with CR vs. CR on MoCA scale. MA, manual acupuncture; EA, electro-acupuncture; CR, cognitive rehabilitation.

**Figure 8:**
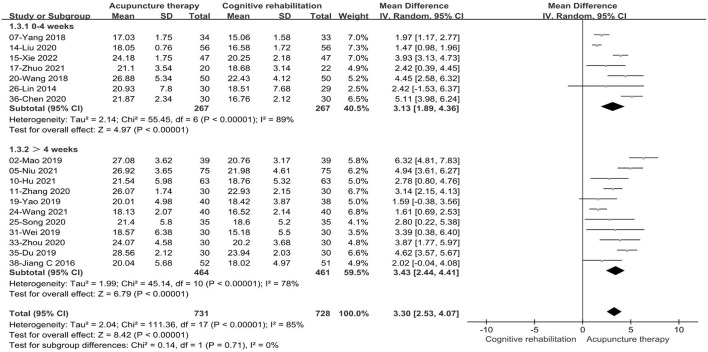
Forest plot of the treatment duration subgroup of acupuncture treatment combined with CR vs. CR on MoCA scale.

We conducted subgroup analysis based on acupuncture modality and treatment duration. There were no statistically significant differences in the comparisons between subgroups (*P* = 0.16, I^2^ = 49.7%, acupuncture modality; *P* = 0.71, I^2^ = 0, treatment duration). In the subgroup analysis of acupuncture types, the combined results favored EA+CR with reduced heterogeneity (MD: 2.17, 95%CI: 0.65–3.70, *P* = 0.005, see Figure 7). Meanwhile, compared to CR alone, MA+CR also improved MoCA scores to a greater extent in patients with PSCI (MD: 3.45, 95%CI:2.54–4.35, *P* < 0.00001, see Figure 7). Furthermore, in the subgroup analysis of treatment duration, we also found that the treatment effect of acupuncture did not change with increasing treatment duration (MD: 3.13, 95%CI: 1.89–4.36, *P* < 0.00001, 0–4 weeks; MD: 3.43, 95%CI:2.44–4.41, *P* < 0.00001, >4weeks; see Figure 8). The results of the sensitivity analysis then showed that excluding any of the studies had little effect on the MD values of the pooled data, and we were unable to find a clear reason for the heterogeneity. Differences in acupoint selection, needle retention time, and treatment frequency among the included studies may be potential causes of heterogeneity.

##### 3.5.1.3. Loewenstein occupational therapy cognitive assessment

**Figure 9:**
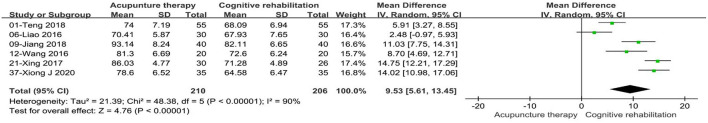
Forest plot of acupuncture therapy combined with CR vs. CR on LOTCA scale.

Sufficient information regarding the LOTCA scale was detected in six studies (32, 37, 40, 43, 52, 68) with 416 PSCI patients. The merged data using the random effects model demonstrated that there was a statistically significant difference between the acupuncture treatment combined with CR and CR alone (MD: 9.53, 95%CI: 5.61–13.45, *P* < 0.00001, see Figure 9). Unfortunately, the results of the sensitivity analysis confirmed little change in MD values for the combined results regardless of the exclusion of any study, while heterogeneity also did not significantly reduce.

#### 3.5.2. Ability to perform activities of daily living

##### 3.5.2.1. Modified barthel index

Seventeen studies (33, 35–37, 39, 40, 43, 47, 49, 51, 52, 55, 56, 58, 59, 65, 67) with 1,295 patients adopted the MBI scale to evaluate patients' ability to perform activities of daily living. Because of high heterogeneity (*P* < 0.00001, I^2^ = 94%), a random effects model was used. The combined data demonstrated that acupuncture treatment combined with CR had a significant advantage over the control group in terms of improving the ability to self-care (MD: 8.66, 95%CI: 5.85–11.47, *P* < 0.00001, see Supplementary Figure 1).

We performed subgroup analysis of the included studies according to acupuncture modality and treatment period. The results of the subgroup analysis based on acupuncture modality showed that MA plus CR (MD: 9.15, 95%CI: 6.17–12.14, *P* < 0.00001, see Supplementary Figure 2) and EA plus CR (MD: 1.74, 95%CI: 0.13–3.35, *P* = 0.03, see Supplementary Figure 2) performed better in improving self-care ability than CR alone. In the subgroup analysis of treatment cycles, we found a significant difference in the treatment effect of acupuncture treatment combined with CR compared with the CR alone (MD: 8.41, 95%CI: 4.55–12.28, *P* < 0.0001, 0–4 weeks; MD: 8.79, 95%CI: 4.59–12.99, *P* < 0.0001, > 4 weeks; see Supplementary Figure 3). One study (59) was the reason for heterogeneity in the 0–4 weeks treatment duration subgroup. It had an unreported mean age and a longer duration of disease compared to other studies, which may have contributed to the heterogeneity. After excluding this study, the MD values of the pooled data still favored acupuncture treatment combined with CR (see Supplementary Table 1). In sensitivity analyses of other subgroups, heterogeneity did not appear significantly reduced regardless of which studies were excluded, while the pooled data did not show significant fluctuations.

##### 3.5.2.2. Functional independence measure

Adequate information regarding the FIM scale was found in three studies (50, 63, 69) with 281 PSCI patients. Because the heterogeneity was acceptable (*P* = 0.26, I^2^ = 26%), a fixed effect model was adopted. The combined data demonstrated significant advantages of acupuncture treatment combined with CR over CR alone in improving the self-care ability in patients with PSCI (MD: 5.24, 95%CI: 3.90–6.57, *P* < 0.00001, see Supplementary Figure 4).

## 4. Adverse events

By scanning 38 studies, we found that the majority of patients who suffered adverse events were able to recover spontaneously without medical intervention. The adverse events are described specifically as follows: one study (69) reported no adverse events occurred during the treatment. Two studies (38, 50) reported five patients who suffered from subcutaneous edema during acupuncture treatment, which was relieved by compression. In addition, one patient experienced dizziness as a result of using acupuncture therapy for the first time and retaining the needle for a long period, which was alleviated by drinking warm water. No adverse events were mentioned in the remaining studies. The merged data using a fixed effects model demonstrated that there was no statistically significant difference between acupuncture treatment and CR regarding the occurrence of adverse effects (RR: 6.83, 95%CI: 0.86–54.13, I^2^ = 0%, *P* = 0.07, see Supplementary Figure 5).

## 5. Publication bias

The funnel plot and Egger's test were adopted to evaluate publication bias based on the MMSE, MoCA and MBI scales. The funnel plot distribution was asymmetric, with some studies outside the 95% confidence interval (see Supplement Figures 6–8). Egger's tests showed that there were publication biases for the MBI scale (*P* = 0.028) but not MMSE (*P* = 0.341) and MoCA (*P* = 0.10) scales (see Supplement Tables 2–4). The results of funnel plot and Egger's test revealed potential publication bias.

## 6. Evidence assessment of outcome measures

The strength of evidence for five scales mentioned above was evaluated as a “low” level of certainty. Deficiencies in the study design and considerable statistical heterogeneity were the main reasons for the downgraded certainty of the evidence (see Table 3).

**Table 3:** Results of evidence assessment.

**Quality assessment**							**No of patients**		**Effect**		**Quality**	**Importance**
**No of studies**	**Design**	**Risk of bias**	**Inconsistency**	**Indirectness**	**Imprecision**	**Other considerations**	**Acupuncture therapy**	**Cognitive rehabilitation**	**Relative (95% CI)**	**Absolute**		
**Mental State Examination Scale (MMSE)**												
25	Randomized trials	Serious^a^	Serious^b^	No serious	Not serious	None	1,049	1,050	-	MD 3.94 higher (3.16 to 4.72 higher)	⊕⊕OO Low	Critical
**Montreal Cognitive Assessment Scale (MoCA)**												
18	Randomized trials	Serious^a^	Serious^b^	No serious	Not serious	None	731	728	-	MD 3.3 higher (2.53 to 4.07 higher)	⊕⊕OO Low	Critical
**Loewenstein Occupational Therapy Cognitive Assessment (LOTCA)**												
6	Randomized trials	Serious^a^	Serious^b^	No serious	Not serious	None	210	206	-	MD 9.53 higher (5.61 to 13.45 higher)	⊕⊕OO Low	Important
**Modified Barthel Index (MBI)**												
17	Randomized trials	Serious^a^	Serious^b^	No serious	Not serious	None	646	649	-	MD 8.66 higher (5.85 to 11.47 higher)	⊕⊕OO Low	Important
**Functional Independence Measure (FIM)**												
3	Randomized trials	Serious^a^	No serious	No serious	Serious^c^	None	142	139	-	MD 5.24 higher (3.9 to 6.57 higher)	⊕⊕OO Low	Important

a Inadequate description of allocation concealment and failure to implement or describe blinding of participants and personnel.

b Considerable statistical heterogeneity.

c Total sample size was < 400.

## 7. Discussion

In this systematic review, 38 RCTs involving a total of 2,971 patients were included. Our findings from this review suggested that the combination of acupuncture treatment and CR was beneficial for global cognitive function (measured by MMSE, MoCA, and LOTCA) and activities of daily living (measured by MBI and FIM) in patients with PSCI. The results of the subgroup analysis showed no difference in the improvement of cognitive function between electro-acupuncture and manual acupuncture (measured by MoCA). Furthermore, we also observed that electro-acupuncture combined with CR did not show sufficient advantages compared to CR alone (measured by MMSE). In terms of treatment duration, the results did not change with increasing treatment duration (measured by MMSE, MoCA, and MBI). Two studies (38, 50) (5.3%) reported AEs associated with acupuncture treatment, with the major AEs including subcutaneous edema and fainting. Sensitivity analysis showed that the effect of acupuncture treatment combined with CR on the cognitive function and daily living abilities of PSCI patients was robust. The existence of methodological flaws and high heterogeneity in the included studies makes the certainty of the evidence was “low” level.

After searching, we found three similar studies (24–26) regarding acupuncture for the treatment of PSCI. The meta-analysis published in 2017 searched the literature up to 2016 and compared electro-acupuncture with cognitive training or pharmacotherapy, eventually including 14 RCTs with a total of 896 patients. The results demonstrated that electro-acupuncture did not show a significant advantage in the improvement of daily living abilities in patients with PSCI, which is contrary to our findings in this review. We observed that only four studies were used to analyze the effect of electro-acupuncture on the ability to perform daily living in patients with PSCI. Considering that there was a deficiency in the sample size of the included studies could be the source of this discrepancy. The merged results of the study may change with increasing sample size, as in our review. Furthermore, the study only assessed the quality of RCT reports regarding electro-acupuncture for PSCI and did not cover other acupuncture types (e.g., body and scalp acupuncture), which also contributed to the shortage in the sample size and limitations of the findings.

Notably, with the continuous deterioration of cognitive function in patients with PSCI, the ability to perform activities of daily living is unavoidably impaired. Consequently, it is particularly important to assess whether PSCI patients' ability to care for themselves improves after receiving acupuncture treatment. Two meta-analyses published in 2020 and 2021 respectively, in which only the MMSE and MoCA scales were selected as outcome indicators to evaluate improvements in cognitive function, reached conclusions consistent with our findings. In contrast, our study additionally added the MBI and FIM scales to observe changes in the self-care ability of daily living in PSCI patients after receiving acupuncture treatment. However, we must take into account that the methodological quality of the RCTs in this meta-analysis is flawed. For example, 10 RCTs (33, 43, 47, 50, 51, 53, 63, 64, 66, 67) did not follow a strict randomization sequence of generation, but only mentioned the term “randomization,” from which we could not determine the exact method of generation. Second, none of the included studies in this meta-analysis mentioned details about allocation concealment. Only three studies (61, 65, 68) achieved blinding of participants, operators, and outcome assessors. Accordingly, the conclusions of this meta-analysis should be treated with caution.

The potential mechanisms of acupuncture for the treatment of PSCI have been extensively studied. The possible mechanisms of PSCI treatment are as follows: Frist, nuclear factor-κB (NF-κB) is an important intracellular transcriptional regulator involved in a range of immune and inflammatory responses. After the ischemic stroke occurs, NF-κB enters the nucleus in an activated state, promoting an inflammatory response that exacerbates brain tissue damage and leads to cognitive impairment (70). Acupuncture can inhibit the entry of NF-κB into the nucleus and reduce the production of inflammatory factors (71). Second, morphological and quantitative damage of synapses is one of the important causes of cognitive impairment in PSCI patients (72). Acupuncture can improve synaptic plasticity by upregulating the expression of postsynaptic mitogen-95 and synaptophysin proteins, resulting in the establishment of effective intracerebral collateral circulation (73). Third, acupuncture can improve neurological deficits in PSCI patients by promoting the expression of brain-derived neurotrophic factors and vascular endothelial growth factors (74). Fourth, acupuncture can improve cognitive function by inhibiting oxidative stress in brain tissue to reduce neuronal cell damage (75).

However, before applying the results to clinical practice, we must take into account some limitations in this review. First, although 38 RCTs were ultimately included in this mate-analysis, the majority of studies suffered from unclear methods of random sequence generation, lack of blinding of participants and operators, and uncertainty in blinding for outcome assessors. Specifically, 26% of the studies did not specify the method of random sequence generation. None of the randomized controlled trials implemented allocation concealment. Eighty-two percent of the trials did not describe blinding of outcome assessors. Eighty-six percent of the trials did not describe the blinding of participants and operators. In addition, only 12 studies were approved by the ethics committee and formally registered. These factors will inevitably lead to some degree of selection bias, detection bias, and reporting bias. Second, another limitation is the presence of a high degree of heterogeneity among the included studies. Although we attempted to find the cause of high heterogeneity through subgroup analysis and sensitivity analysis, acupuncture, as a complex treatment, may itself be a source of heterogeneity. To derive maximum benefit from acupuncture treatment, we need to consider a combination of factors, such as the selection and combination of acupoints, the depth and retention time of needles, and the duration and frequency of treatment. Although we grouped the studies by acupuncture modality and treatment period, these parameters are still variable and we were unable to precisely and reliably identify the cause of the significant heterogeneity. Therefore, future experimental studies should elaborate on the acupuncture protocol to further improve the integrity of the report. Furthermore, variability in the skill level of acupuncture therapists can also have an impact on treatment outcomes. The variability of these factors may account for the high degree of heterogeneity. These two limitations are the main reasons for the lower level of evidence in this review. In addition, most of the studies in this review were published in Chinese, and only two studies were published in English databases. Therefore, the conclusions drawn need to be interpreted with caution as the superior efficacy of acupuncture over cognitive rehabilitation in the treatment of PSCI in China. Patients with PSCI have a long-term recovery period, but the studies included all used short-term outcome indicators and lacked long-term follow-up of patients. Therefore, the long-term efficacy of acupuncture for PSCI needs to be further explored. In addition, we need to note that in this review, outcome indicators such as MMSE, MoCA, and LOTCA are presented as cognitive screening tools for patients with PSCI, rather than serving as comprehensive cognitive assessments. A further limitation is that the severity of cognitive impairment in patients with PSCI was not clearly defined in this study in terms of inclusion criteria. Considering that PSCI is a disease with highly complex features and the presence of other comorbidities that can affect the cognitive function of patients to varying degrees, this may also contribute to the high degree of heterogeneity. Therefore, it is suggested that future studies could be conducted in specific areas to provide more definitive and high-quality evidence for the efficacy of acupuncture in the treatment of PSCI. Finally, of the 38 studies included, one study reported no AEs during acupuncture treatment and two studies reported mild AEs in six patients. Acupuncture treatment appears to be relatively safe and did not cause severe AEs.

Because of the unique geographical characteristics of acupuncture treatment, we had to search as comprehensively as possible for RCTs related to acupuncture treatment in Chinese journals. However, we noticed that the vast majority of studies published in Chinese databases had experimental design flaws. To truly and effectively evaluate the efficacy of acupuncture for PSCI and to provide high-quality evidence for clinical practice, future studies need to be more rigorous in terms of experimental design and methodology. As a non-pharmaceutical type of intervention, acupuncture therapists require the selection of different acupoints for treatment in clinical practice, which makes it difficult to achieve operator blinding. However, we can achieve blinding of participants through sham acupuncture or placebo acupuncture to minimize the occurrence of bias. Also, blinding of outcome assessors is feasible and necessary. In addition, 68%of the studies in this review were not pre-registered. Therefore, future study protocols should be prospectively registered in advance in registries to reduce the possibility of reporting bias.

## 8. Conclusion

This systematic review suggests that the addition of acupuncture therapy to cognitive rehabilitation may have a positive effect on improving cognitive function and activities of daily living in patients with PSCI. However, due to the methodological limitations of the included studies, this evidence was rated as “low” by GRADE, and the results should be treated with caution. Future clinical studies should use a high-quality randomized, double-blind controlled trial design. Meanwhile, long-term follow-up and efficacy assessment as well as large multicenter sample size clinical studies are highly desirable.

## Data availability statement

The original contributions presented in the study are included in the article/Supplementary material, further inquiries can be directed to the corresponding author.

## Author contributions

FY-C proposed the review, designed the protocol, made further revisions, and refinements to the article. ZK and HH-Z performed the literature search and screening. XP-L and BQ-W performed the data extraction and tabulation. JQ-H and PQ completed the assessment of the risk of bias and evidence rating. YL and LZ analyzed the data and completed the initial manuscript. All authors read and approved the final version of the manuscript for acceptance for publication.
