# Supplementary material for: Acupuncture treatment vs. cognitive rehabilitation for post-stroke cognitive impairment: A systematic review and meta-analysis of randomized controlled trials
Source: Front Neurol. 2023 Feb 9;14:1035125. doi: 10.3389/fneur.2023.1035125 (PMC9946978; doi:10.3389/fneur.2023.1035125)
Supplement: Supplementary file 1 [file Data_Sheet_1.docx]

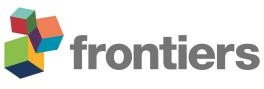


***Supplementary Material***

Acupuncture Treatment Versus Cognitive Rehabilitation for Post-Stroke Cognitive Impairment: A Systematic Review and Meta-Analysis of Randomized Controlled Trials

Yang Liu^1,2^, Xingping-Li^1,2^, Jiangqin-Han^1,2^, Zi Ke^1,2^, Honghang-Zhu^1,2^, Fuyan-Chen^1,2, *^

*^1^Department of acupuncture. First Teaching Hospital of Tianjin University of Traditional Chinese Medicine, Anshanxi Road, Nankai District, 300193 Tianjin, China*

*^2^National Clinical Research Center for Chinese Medicine Acupuncture and Moxibustion, China*

**Appendix:** search strategies for databases

**PubMed search strategy**

| Strategies |  |
| --- | --- |
| #1 | "Acupuncture"[Mesh] OR "Acupuncture Therapy"[Mesh] OR "Acupuncture Points"[Mesh] |
| #2 | “Acupuncture” [Title/Abstract]) OR (Acupuncture Therapy [Title/Abstract])) OR (Acupuncture Points [Title/Abstract])) OR (Electroacupuncture [Title/Abstract])) OR (Acupuncture ear [Title/Abstract])) OR (Scalp acupuncture [Title/Abstract] |
| #3 | (#1 OR #2) |
| #4 | Cognitive Dysfunction [Mesh] |
| #5 | “Cognitive dysfunction” [Title/Abstract]) OR (cognitive impairment [Title/Abstract])) OR (cognitive decline [Title/Abstract])) OR (mental deterioration [Title/Abstract])) OR (PSCI[Title/Abstract])) OR (aged-related memory disorders [Title/Abstract] |
| #6 | (#4 OR #5) |
| #7 | "Stroke"[Mesh] OR "Stroke, Lacunar"[Mesh] OR "Hemorrhagic Stroke"[Mesh] OR "Embolic Stroke"[Mesh] OR "Thrombotic Stroke"[Mesh] OR "Ischemic Stroke"[Mesh] |
| #8 | stroke[Title/Abstract]) OR (stroke, lacunar [Title/Abstract])) OR (hemorrhagic stroke[Title/Abstract])) OR (embolic stroke[Title/Abstract])) OR (thrombotic stroke[Title/Abstract])) OR (ischemic stroke[Title/Abstract])) OR (cerebrovascular accident[Title/Abstract])) OR (cerebrovascular apoplexy[Title/Abstract])) OR (apoplexy[Title/Abstract])) OR (lacunar stroke[Title/Abstract])) OR (lacunar syndrome[Title/Abstract])) OR (lacunar infarction[Title/Abstract])) OR (intracerebral hemorrhagic stroke[Title/Abstract])) OR (intracerebral hemorrhage stroke[Title/Abstract])) OR (acute ischemic stroke[Title/Abstract] |
| #9 | (#7 OR #8) |
| #10 | (#3 AND #6 AND #9) |

**Web of science search strategy**

(TS=(acupuncture OR acupuncture therapy OR electroacupuncture OR acupuncture ear OR acupuncture points OR scalp acupuncture)) ADN TS=(cognitive dysfunction OR cognitive impairment OR cognitive decline OR mental deterioration OR PSCI OR aged-related memory disorders) AND TS=(stroke OR stroke, lacunar OR hemorrhagic stroke OR embolic stroke OR thrombotic stroke OR ischemic stroke OR cerebrovascular accident OR cerebrovascular apoplexy OR apoplexy OR lacunar stroke OR lacunar syndrome OR lacunar infarction OR intracerebral hemorrhagic stroke OR intracerebral hemorrhage stroke OR acute ischemic stroke)

**Embase search strategy**

(acupuncture:ab,ti OR 'acupuncture therapy':ab,ti OR electroacupuncture:ab,ti OR 'acupuncture ear':ab,ti OR 'acupuncture points':ab,ti OR 'scalp acupuncture':ab,ti) AND ('cognitive dysfunction':ab,ti OR 'cognitve impairment':ab,ti OR 'cognitive decline':ab,ti OR 'mentai deterioration':ab,ti OR psci:ab,ti OR 'aged-related memory disorders':ab,ti) AND (stroke:ab,ti OR stroke,lacunar:ab,ti OR 'hemorrhagic stroke':ab,ti OR 'embolic stroke':ab,ti OR 'thrombotic stroke':ab,ti OR 'ischemic stroke':ab,ti OR 'cerebrovascular accident':ab,ti OR 'cerebrovascular apoplexy':ab,ti OR apoplexy:ab,ti OR 'lacunar stroke':ab,ti OR 'lacunar syndrome':ab,ti OR 'lacunar infarction':ab,ti OR 'intracerebral hemorrhagic stroke':ab,ti OR 'intracerebral hemorrhage stroke':ab,ti OR 'acute ischemic stroke':ab,ti)

**Cochrane library search strategy**

(acupuncture OR acupuncture therapy OR electroacupuncture OR acupuncture ear OR acupuncture points OR scalp acupuncture):ti,ab,kw AND (cognitive dysfunction OR cognitive impairment OR cognitive decline OR mental deterioration OR PSCI OR aged-related memory disorders):ti,ab,kw AND (stroke OR stroke, lacunar OR hemorrhagic stroke OR embolic stroke OR thrombotic stroke OR ischemic stroke OR cerebrovascular accident OR cerebrovascular apoplexy OR apoplexy OR lacunar stroke OR lacunar syndrome OR lacunar infarction OR intracerebral hemorrhagic stroke OR intracerebral hemorrhage stroke OR acute ischemic stroke):ti,ab,kw

Chinese Biological Medicine Database search strategy

1 "针灸疗法"[不加权:扩展] OR "针刺疗法"[不加权:扩展] OR "毫针"[不加权:扩展]

2"针刺疗法"[全部字段:智能] OR "针灸疗法"[全部字段:智能] OR "毫针"[全部字段:智能] OR "电针"[全部字段:智能] OR "头针"[全部字段:智能] OR "耳针"[全部字段:智能] OR "体针"[全部字段:智能] OR "针灸"[全部字段:智能] OR "针刺"[全部字段:智能]

3 (#1) OR (#2)

4 "卒中"[不加权:扩展]) OR "中风"[不加权:扩展]) OR "脑血管障碍"[不加权:扩展]

5 "中风"[全部字段:智能] OR "卒中"[全部字段:智能] OR "脑栓塞"[全部字段:智能] OR "脑卒中"[全部字段:智能] OR "脑梗塞"[全部字段:智能] OR "脑梗死"[全部字段:智能] OR "脑出血"[全部字段:智能] OR "脑血管意外"[全部字段:智能] OR "脑血管病"[全部字段:智能]

6 (#4) OR (#5)

7"认知障碍"[不加权:扩展]

8"认知障碍"[全部字段:智能] OR "认知功能障碍"[全部字段:智能] OR "认知损害"[全部字段:智能] OR "认知损伤"[全部字段:智能] OR "神经行为障碍"[全部字段:智能]) OR (("认知障碍"[不加权:扩展]

9 (#4) OR (#8)

10 (#3) AND (#6) AND (#9)

China National Knowledge Infrastructure (CNKI) database search strategy

（主题：针灸疗法 + 针灸疗法 +毫针（精确）） OR （篇摘要：针刺疗法 + 针灸疗法 + 毫针 + 体针 + 头针 + 电针 + 针灸 + 针刺（精确）） AND （主题：中风 + 卒中 + 脑血管障碍（精确）） OR （篇摘要：中风 + 卒中 +脑血管障碍 + 脑梗塞 + 脑梗死 + 脑出血 + 脑卒中 + 脑栓塞 + 脑血管病 + 脑血管意外（精确）） AND （主题：认知障碍（精确）） OR （篇关摘：认知障碍 + 认知功能障碍 + 认知损伤 + 认知损害 + 神经行为障碍（精确））

Chinese Science and Technology periodical Database (VIP) search strategy

（题名或关键词=针刺疗法 OR 针灸疗法 OR 毫针 OR 电针 OR 体针 OR 头针 OR 耳针 OR 针刺 OR 针灸) AND （题名或关键词=卒中 OR 中风 OR 脑血管障碍 OR 脑卒中 OR 脑梗死 OR 脑梗塞 OR 脑出血 OR 脑栓塞 OR 脑血管病 OR 脑血管病意外) AND （题名或关键词=认知障碍 OR 认知功能障碍 OR 认知损害 OR 认知损伤 OR 神经行为障碍）

Wan fang Database search strategy

题名或关键词：（针刺疗法 OR 针灸疗法 OR 毫针 OR 电针 OR 体针 OR 头针 OR 耳针 OR 针刺 OR 针灸） AND 题名或关键词：（卒中 OR 中风 OR 脑血管障碍 OR 脑卒中 OR 脑梗死 OR 脑梗塞 OR 脑出血 OR 脑栓塞 OR 脑血管病 OR 脑血管病意外） AND 题名或关键词：（认知障碍 OR 认知功能障碍 OR 认知损害 OR 认知损伤 OR 神经行为障碍）
