# Supplementary material for: Acupuncture treatment vs. cognitive rehabilitation for post-stroke cognitive impairment: A systematic review and meta-analysis of randomized controlled trials
Source: Front Neurol. 2023 Feb 9;14:1035125. doi: 10.3389/fneur.2023.1035125 (PMC9946978; doi:10.3389/fneur.2023.1035125)
Supplement: Supplementary file 2 [file Data_Sheet_2.docx]

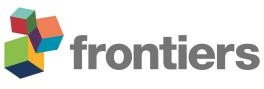


***Supplementary Material***

Acupuncture Treatment Versus Cognitive Rehabilitation for Post-Stroke Cognitive Impairment: A Systematic Review and Meta-Analysis of Randomized Controlled Trials

Yang Liu^1,2^, Xingping-Li^1,2^, Jiangqin-Han^1,2^, Zi Ke^1,2^, Honghang-Zhu^1,2^, Fuyan-Chen^1,2, *^

*^1^Department of acupuncture. First Teaching Hospital of Tianjin University of Traditional Chinese Medicine, Anshanxi Road, Nankai District, 300193 Tianjin, China*

*^2^National Clinical Research Center for Chinese Medicine Acupuncture and Moxibustion, China*





**Supplementary Figure 1.** Forest plot of acupuncture treatment combined with CR versus CR on MBI





**Supplementary Figure 2** Forest plot of the intervention subgroup of acupuncture treatment combined with CR versus CR on MBI





**Supplementary Figure 3** Forest plot of the treatment duration subgroup of acupuncture treatment combined with CR versus CR on MBI

**
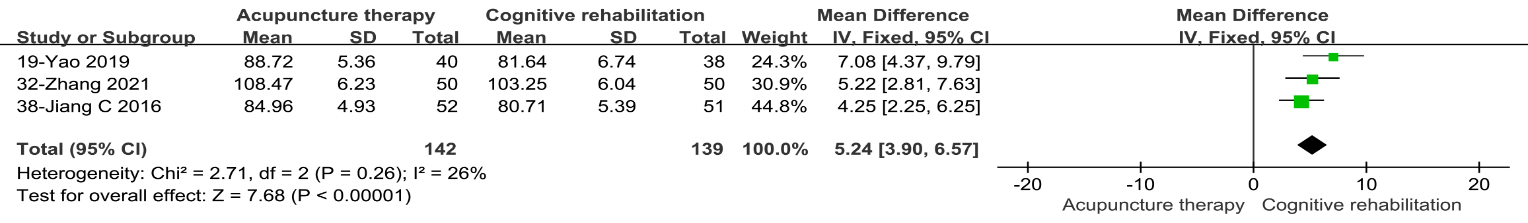
**

**Supplementary Figure 4.** The forest plot of acupuncture treatment combined with CR versus CR on FIM


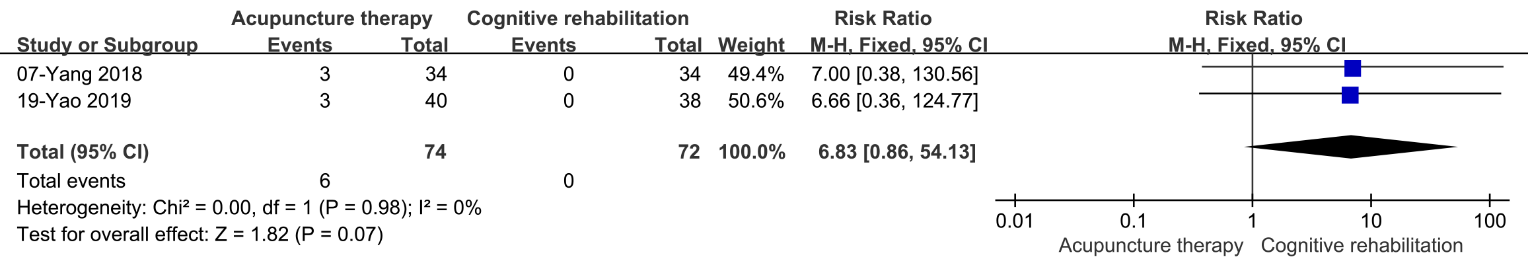


**Supplementary Figure 5** The forest plot of acupuncture treatment combined with CR versus CR on the incidence of adverse events

##

Supplementary Material



**Supplementary Figure 6.** The funnel plot of MMSE

**Supplementary Figure 7.** The funnel plot of MoCA

## Supplementary Material

**

**

**Supplementary Figure 8.** The funnel plot of MBI
