# Supplementary material for: Acupuncture treatment vs. cognitive rehabilitation for post-stroke cognitive impairment: A systematic review and meta-analysis of randomized controlled trials
Source: Front Neurol. 2023 Feb 9;14:1035125. doi: 10.3389/fneur.2023.1035125 (PMC9946978; doi:10.3389/fneur.2023.1035125)
Supplement: Supplementary file 3 [file Data_Sheet_3.docx]

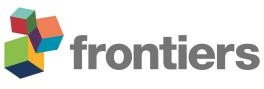


***Supplementary Material***

Acupuncture Treatment Versus Cognitive Rehabilitation for Post-Stroke Cognitive Impairment: A Systematic Review and Meta-Analysis of Randomized Controlled Trials

Yang Liu^1,2^, Xingping-Li^1,2^, Jiangqin-Han^1,2^, Zi Ke^1,2^, Honghang-Zhu^1,2^, Fuyan-Chen^1,2, *^

*^1^Department of acupuncture. First Teaching Hospital of Tianjin University of Traditional Chinese Medicine, Anshanxi Road, Nankai District, 300193 Tianjin, China*

*^2^National Clinical Research Center for Chinese Medicine Acupuncture and Moxibustion, China*

**Supplementary Table 1 Results of the sensitivity analyses**

| **Study** | **Subgroup** | **MD (95%CI)** | **Tau^2^** | **I^2^** | **P** |
| --- | --- | --- | --- | --- | --- |
| MMSE | | | | | |
| Niu 2021 | > 4 weeks | 3.08 (2.55, 3.62) | 0.47 | 52% | 0.01 |
| MBI | | | | | |
| Zeng 2018 | 0-4 weeks | 9.73 (7.62, 11.84) | 2.15 | 38% | 0.17 |

**Supplementary Table 2. Tests for Publication Bias of MMSE (Egger’s test)**

| Std_Eff | Coef. | Std. Err. | t | P>\|t\| | [95% Conf. Interval] | |
| --- | --- | --- | --- | --- | --- | --- |
| Slope | 5.11089 | 1.034706 | 4.94 | 0.00 | 2.970438 | 7.251341 |
| bias | -1.575534 | 1.620248 | -0.97 | 0.341 | -4.927272 | 1.776204 |

**Supplementary Table 3. Tests for Publication Bias of MoCA (Egger’s test)**

| Std_Eff | Coef. | Std. Err. | t | P>\|t\| | [95% Conf. Interval] | |
| --- | --- | --- | --- | --- | --- | --- |
| Slope | 1.789961 | .6779622 | 2.64 | 0.018 | .3527452 | 3.227176 |
| bias | 2.040283 | 1.170817 | 1.74 | 0.10 | -.4417381 | 4.522303 |

**Supplementary Table 4. Tests for Publication Bias of MBI (Egger’s test)**

| Std_Eff | Coef. | Std. Err. | t | P>\|t\| | [95% Conf. Interval] | |
| --- | --- | --- | --- | --- | --- | --- |
| Slope | 1.205165 | 2.323634 | 0.52 | 0.612 | -3.747545 | 6.157874 |
| bias | 4.140961 | 1.696738 | 2.44 | 0.028 | .5244501 | 7.757472 |
